# Effects of 3-beta-diol, an androgen metabolite with intrinsic estrogen-like effects, in modulating the aquaporin-9 expression in the rat efferent ductules

**DOI:** 10.1186/1477-7827-4-51

**Published:** 2006-10-06

**Authors:** Patrícia Picciarelli-Lima, André G Oliveira, Adelina M Reis, Evanguedes Kalapothakis, Germán AB Mahecha, Rex A Hess, Cleida A Oliveira

**Affiliations:** 1 Department of Morphology, Federal University of Minas Gerais, Cx. Postal 486, CEP 31.270-901, Belo Horizonte, Minas Gerais, Brazil; 2 Department of Physiology and Biophysics, Federal University of Minas Gerais, Cx. Postal 486, CEP 31.270-901, Belo Horizonte, Minas Gerais, Brazil; 3 Department of General Biology, Federal University of Minas Gerais, Cx. Postal 486, CEP 31.270-901, Belo Horizonte, Minas Gerais, Brazil; 4 Department of Veterinary Biosciences, University of Illinois, 2001 S. Lincoln, Urbana, IL 61802, USA

## Abstract

**Background:**

Fluid homeostasis is critical for normal function of the male reproductive tract and aquaporins (AQP) play an important role in maintenance of this water and ion balance. Several AQPs have been identified in the male, but their regulation is not fully comprehended. Hormonal regulation of AQPs appears to be dependent on the steroid in the reproductive tract region. AQP9 displays unique hormonal regulation in the efferent ductules and epididymis, as it is regulated by both estrogen and dihydrotestosterone (DHT) in the efferent ductules, but only by DHT in the initial segment epididymis. Recent data have shown that a metabolite of DHT, 5-alpha-androstane-3-beta-17-beta-diol (3-beta-diol), once considered inactive, is also present in high concentrations in the male and indeed has biological activity. 3-beta-diol does not bind to the androgen receptor, but rather to estrogen receptors ER-alpha and ER-beta, with higher affinity for ER-beta. The existence of this estrogenic DHT metabolite has raised the possibility that estradiol may not be the only estrogen to play a major role in the male reproductive system. Considering that both ER-alpha and ER-beta are highly expressed in efferent ductules, we hypothesized that the DHT regulation of AQP9 could be due to the 3-beta-diol metabolite.

**Methods:**

To test this hypothesis, adult male rats were submitted to surgical castration followed by estradiol, DHT or 3-beta-diol replacement. Changes in AQP9 expression in the efferent ductules were investigated by using immunohistochemistry and Western blotting assay.

**Results:**

Data show that, after castration, AQP9 expression was significantly reduced in the efferent ductules. 3-beta-diol injections restored AQP9 expression, similar to DHT and estradiol. The results were confirmed by Western blotting assay.

**Conclusion:**

This is the first evidence that 3-beta-diol has biological activity in the male reproductive tract and that this androgen metabolite has estrogen-like activity in the efferent ductules, whose major function is the reabsorption of luminal fluid.

## Background

Fluid homeostasis is critical for normal function of the male reproductive tract and aquaporins (AQP) play an important role in maintenance of this water and ion balance [1-3]. Some of these integral membrane proteins allow transcellular movement of water, a function associated with their name, but other AQPs permit the movement of small solutes [4]. At least 13 mammalian isoforms (AQP0-AQP12) have been identified, particularly in fluid-transporting tissues, but also in cells not recognized for this activity [5-7]. Several AQPs are expressed in the male genital tract, with specific isoforms identified in testes, efferent ductules, the epididymis and vas deferens [8-12].

The efferent ductules are a major site for fluid homeostasis, as they reabsorb more than 95% of the luminal fluid released from the seminiferous epithelium [13]. These small coiled ductules transport sperm from the rete testis and play a major role in concentrating sperm prior to their maturation in the epididymis [14,15]. The male excurrent ductal epithelia express at least four AQPs, with 1, 9 and 10 found in the efferent ductules and 1,3, and 9 reported in the epididymis [9-11,16-18]. Although AQP1 and 9 are common to both the efferent ductules and epididymis, regulation of their expression shows significant divergence, as AQP1 appears to be constitutively expressed, while AQP9 responds to both estrogen and androgen [16]. Of particular note, testosterone alone was not capable of restoring AQP9 after castration or efferent ductule ligation [16,19]. However, both estradiol and dihydrotestosterone (DHT) restored AQP9 expression in the efferent ductules, but in the initial segment epididymis, only DHT restored AQP9 to normal levels [16].

The differential regulation of AQP9 in the male tract, depending on the steroid hormone and location in the male reproductive tract, suggested that the selective presence of estrogen receptors (ERα and ERβ) [2,15] in efferent ductule and epididymal epithelium and a selective presence of 5α-reductase and DHT activity in maintaining epididymal structure and function [20,21] may be responsible for this unique hormonal activity. Recent data have shown that DHT may be converted into 5α-androstane-3β-17β-diol (3β-diol) in a virtually irreversible reaction [22]. Once considered inactive [23,24], 3β-diol is present in high concentrations in the male and indeed has biological activity [25-30]. However, 3β-diol does not bind to the androgen receptor (AR), but rather to ERα and ERβ, with higher affinity for ERβ [26,31,32]. Based upon these findings, we hypothesized that the modulation of AQP9 by DHT could be indirectly mediated by 3β-diol. To test this hypothesis in the present study, surgical castration followed by hormonal replacement with 3β-diol was performed to investigate the regulation of AQP9 expression in rat efferent ductules. Our results show that the 3β-diol metabolite restores AQP9 immunostaining, similar to DHT and estradiol replacement.

## Methods

### Animals

The present study was performed in adult male 120-day-old Wistar rats, obtained from multiple litters and housed in the Animal Facility (CEBIO) at the Instituto de Ciências Biológicas, Universidade Federal de Minas Gerais – Brazil. The rats were maintained under constant light cycle (12 h of light and 12 h of darkness) and temperature (22°C), and received peletized chow as diet (Nuvital Nutrientes S.A, Colombo, Brazil) and water *ad libitum*. The principles of research involving animals followed the protocol, published by the Universidade Federal de Minas Gerais . The experimental procedures were approved by the institutional Ethical Committee for Animal Experimentation of the Federal University of Minas Gerais (CETEA), Brazil.

### Surgical castration

Adult rats were anaesthetized with intraperitoneal injection of sodium pentobarbital (50 mg/kg) (Cristália Ltda., Itapira, Brazil) and ketamine chloridrate (10 mg/kg) (Laboratorios König S.A., Avellaneda, Argentina). The bilateral castration was performed following the protocol previously described [33]. In summary, the testes-epididymis were exposed through a mid-line ventral scrotal incision, the extratesticular rete testis together with the pampiniform plexus vessels were ligated, the testes were removed, and then the ligated efferent ductules and epididymis were returned into the scrotum. For sham operation, the testes were exposed, manipulated and then reinserted intact into the scrotum. After surgery, the scrotal incision was closed by suture. The animals received the analgesic and anti-inflamatory Banamine^® ^(Schering-Plough S.A., Rio de Janeiro, Brazil) (1.5 mg/kg). Post-operative conditions of the animals were monitored daily.

### Hormone replacement

Starting on the day of bilateral surgical castration, the rats were subjected to hormonal replacement, using subcutaneous injections once per day for 6 days. The treatment period was chosen considering that at this time both circulating and luminal source of hormones as well as sperm passing through the male tract has already been depleted [34-36]. Rats were randomly assigned to one of the following treatment groups (n = 5 for each group): 5 mg of 5α-dihydrotestosterone (DHT – Sigma, St.Louis, USA); 400 μg of 17-β-estradiol-3-benzoate (E2 – Sigma, St.Louis, USA); 3 mg of 5α-androstane-3β-17β-diol (3β-diol – Sigma, St.Louis, USA). Hormone dosages were based on prior studies [26,33]. Steroids dissolved in corn oil were injected with a total volume of 0.1 ml. The same volume of vehicle was given to the castration control group.

### Hormone measurements

Blood samples were collected by cardiac puncture immediately after the animals reached a surgical plane of anesthesia. The plasma was separated by centrifugation and stored at -20°C for subsequent hormone assays. Plasma testosterone and estradiol were measured by radioimmunoassay, using commercial kits (Testosterone MAIA and Estradiol MAIA kits – Adaltis, Rome, Italy), according to instruction of the manufacturer [37]. The limit of testosterone detection was 0.07 ng/ml and the assay coefficient of variation was 4.7%. The antibody used for testosterone radioimmunoassay (RIA) has low cross reactivity to DHT (5%) and other androgens (less than 0.01%). The assay detection limit for estradiol was 15 pg/ml and the intra- and inter-assay coefficient of variation was 4.3% and 5.6%, respectively. All samples were measured in duplicate.

### Tissue preparation

Rats surgically castrated were euthanized 6 days after the initiation of hormone replacement. The rats were anaesthetized (intraperitoneal sodium pentobarbital; 80 mg/kg body weight plus ketamine cloridrate 10 mg/kg body weight), weighed and perfused intracardially with 10% (v/v) neutral buffered formalin (NBF). After fixation, the efferent ductules together with the epididymis, ventral prostate, and seminal vesicle together with coagulating gland were dissected out and weighed. The relative organ weights were calculated per 100 g of body weight and the results expressed as mean ± standard deviation. After weighting, the efferent ductules were dissected out from the epididymis, embedded in paraffin, sectioned (5 μm) and mounted on glass slides.

### Immunohistochemistry

Changes in the expression of AQP9 were investigated by immunohistochemistry following the protocol previously described [16]. Briefly, sections were deparaffinized, rehydrated, and then blocked for endogenous peroxidase. After antigen retrieval using a standard microwave method, the sections were incubated in 10% (v/v) normal goat serum to block nonspecific binding, and then overnight with the AQP9 polyclonal rabbit anti-rat primary antibody (Alpha Diagnostic International, San Antonio, USA), diluted 1:1000. For negative control, phosphate buffer saline (PBS) was used in place of the primary antibody. Biotinylated goat anti-rabbit secondary antibody (Dako, Carpinteria, USA) was used at 1:100 dilution. The labeling was visualized using avidin-biotin complex (Vectastain Elite ABC kit; Vector Laboratories, Burlingame, USA), followed by incubation in 0.05% (w/v) 3,3'diaminobenzidine containing 0.01% (v/v) H_2_O_2 _in 0.05 M Tris/HCl buffer, pH 7.6, until a brown reaction was observed by microscopy. The reaction was stopped by immersion in distilled water. Sections were counterstained with Mayer's haematoxylin then dehydrated in ethanol, cleared in xylene and mounted. For semiquantification of the immunostaining, tissue sections from control and all other experimental groups were run in parallel and staining was performed in triplicate sets to confirm results.

### Semiquantitative immunohistochemical study

AQP9 immunostaining intensity was quantified by computer-assisted image analysis, based on previously reported protocols [19,38]. Pictures from 10 different areas of the proximal region of the efferent ductule epithelium of each animal were taken by using a x40 objective lens of a Nikon Eclipse E600 microscope (Nikon Corp., Melville, USA) and a Nikon Coolpix digital camera (Nikon Corp., Melville, USA). Digital images were processed with Adobe Photoshop (Adobe Systems, Mountain View, USA), converted to the grayscale mode and inverted. The images were then exported to Image-Tool software (version 3.00; University of Texas Health Sciences Center, San Antonio, USA), for quantitative analysis. For this proposal, the stained apical areas of the nonciliated epithelial cells were traced and measured and pixel intensity was determined for the traced areas. Background intensity was determined by tracing an unlabeled area adjacent to the measured cells and subtracted from values detected in the labeled regions. Data are expressed as mean ± standard deviation.

### Western blotting analysis

Efferent ductules of rats (n = 04) which were sham-operated or castrated and submitted to hormonal replacement, as described above, were used for Western blotting assay. Following dissection, the efferent ductules were trimmed of fat tissue, rinsed vigorously in PBS and frozen in liquid nitrogen. After maceration using dry ice, tissues (100 mg) were solubilized in 750 μl of buffer sample (1% sodium dodecyl sulfate, 30 mM Tris-HCl pH 6.8, 2-mercaptoethanol, 12% (v/v) glycerol and bromophenol blue), boiled for 5 min and then subjected to continuous electrophoresis using 12.5% SDS-PAGE (sodium dodecyl sulfate polyacrylamide gel electrophoresis). The separated proteins were transferred to nitrocellulose membrane and blocked with 10% normal goat serum for 1 h at room temperature. Then, the membrane was incubated with rabbit anti-rat primary antibody (alpha Diagnostic International, San Antonio, USA), diluted 1:1000, for 1 h. After washing with PBS-Tween 0.05%, the blot was incubated in goat anti-rabbit secondary antibody diluted 1:1000. After several washes, the reaction was developed by the addition of 0.1% 3,3'diaminobenzidine in PBS containing 0.05% chloronaphtol, 16.6% methanol and 0.04% H_2_O_2_. The reaction was stopped with deionized water.

The AQP9 density was estimated using Scion Image software , as previously described [39]. Briefly, the Western blots were electronically scanned and saved as TIFF image. Specific bands were traced and measured with a constant measurement area around the protein bands. Background intensity was determined by tracing an unlabeled area adjacent to the measured band. The final intensity was determined by subtracting background intensity from positive values for each band.

### Statistical analysis

Treatment related effects in body and organ weights, as well as hormone levels and immunoquantification of AQP9 expression, were analyzed statistically using multiple variance analyses (ANOVA). The post-hoc Student-Newman-Keuls test was used for multiple comparisons between groups. Differences were considered significant at p ≤ 0.05.

## Results

### Plasma hormone levels

**Figure 1:**
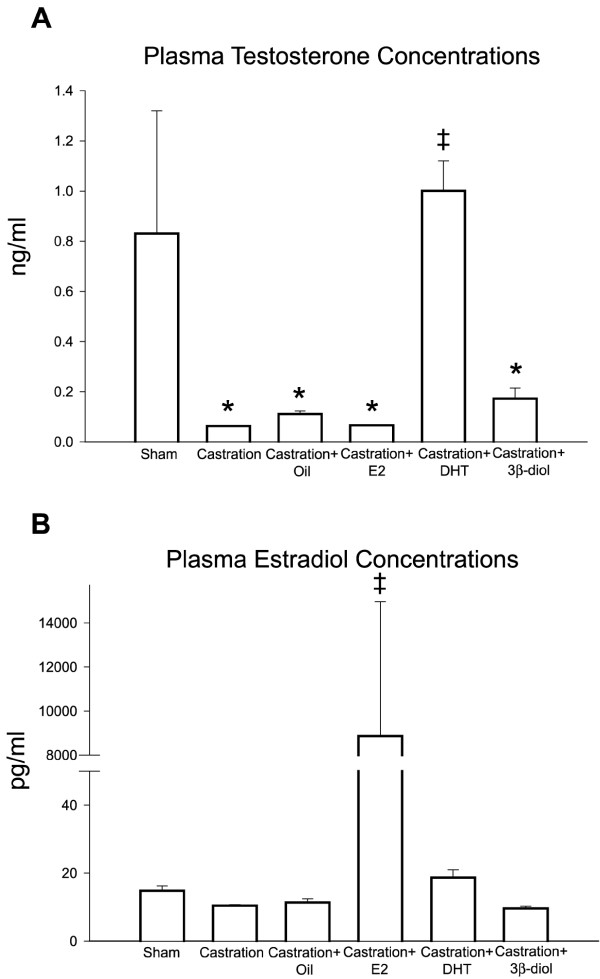
**Plasma hormonal levels after castration and hormonal replacement**. **(A) **Castration resulted in a significant reduction of 93% in the serum testosterone concentration. Corn oil, estradiol (E2) and 3β-diol maintained the testosterone level similar to castrated rat, while DHT increased the plasma testosterone concentration. **(B) **The plasma estradiol concentration was not affected by the castration or replacement with corn oil, DHT and 3β-diol. However, after estradiol replacement, a significant increase in the plasma estradiol level was observed. Values represent mean ± SEM; n = 5 in each group; (*) = P ≤ 0,05 when compared to control group; (‡) = P ≤ 0,05 when compared to castrated group.

Surgical castration resulted in a dramatic reduction of 93% in the serum testosterone concentration. DHT treatment returned testosterone concentrations to control levels. In contrast, estradiol and 3β-diol treatments showed no effect on testosterone levels (Fig. 1).

Plasma estradiol concentrations were not affected by the surgical castration procedure. DHT and 3β-diol, as well as the corn oil vehicle injections showed no effect on the hormone levels, compared to intact controls; however, estradiol treatments resulted in a significant increase in plasma estradiol levels (Fig. 1).

### Body and organ weights

**Figure 2:**
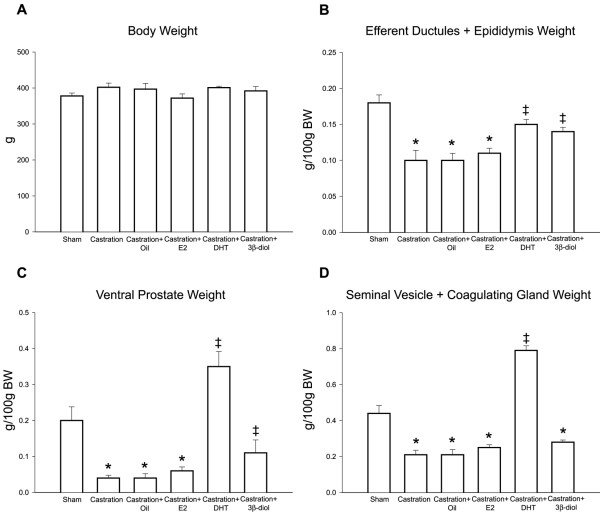
**Body, epididymis and sexual accessory gland weights after castration and hormonal replacement**. **(A) **No significant difference in body weight was observed in any experimental group.**(B) **Castration significantly diminished the efferent ductules/epididymis relative weights, whereas replacement with DHT and 3β-diol, but not estradiol, recovered the organ weight. **(C) **The weight of ventral prostate were significantly decreased after castration, but restored by DHT and 3β-diol. **(D) **There was also a significant reduction in the seminal vesicle/coagulating gland weight, which was recovered by DHT, but not by 3β-diol or estradiol. Values represent mean ± SEM; n = 5 in each group; (*) = P ≤ 0,05 when compared to control group; (‡) = P ≤ 0,05 when compared to castrated group.

No significant difference in body weight was observed when experimental groups were compared to control. On the other hand, castration significantly diminished the efferent ductules/epididymis (44%), ventral prostate (80%) and seminal vesicle/coagulating gland (52%) relative weights (Fig. 2). Treatment with DHT, but not estradiol, was able to restore weights of accessory sex glands and the efferent ductules/epididymis. Compared to castrated animals, a slight but significant increase in the weights of the epididymis (P = 0.048) and ventral prostate (P = 0.005), but not the seminal vesicle, occurred after the 3β-diol replacement.

### AQP9 expression

**Figure 3:**
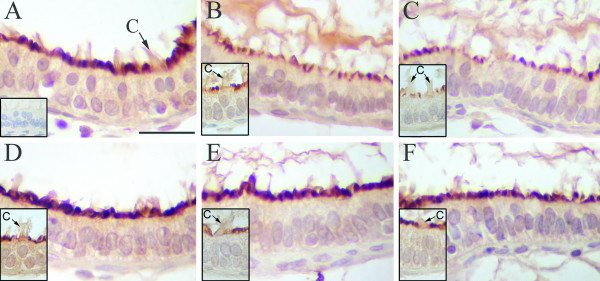
**Expression of AQP9 in the rat efferent ductules**. (**A**) In control rats AQP9 was expressed in the microvillus border of the non-ciliated cells in the epithelium. Ciliated cells (c) were negative for AQP9. (**B**) Castration caused an evident decrease in the AQP9 staining when compared to controls. (**C**) Treatment with corn-oil maintained the AQP9 staining at levels similar to castrated rats. Replacement with estradiol (**D**), DHT (**E**) and 3β-diol (**F**) recovered the epithelial AQP9 staining to control levels. Insert in A = negative control. Inserts in B-F = detail of ciliated cells negative for AQP9. Scale bar in A = 25 μm.

**Figure 4:**
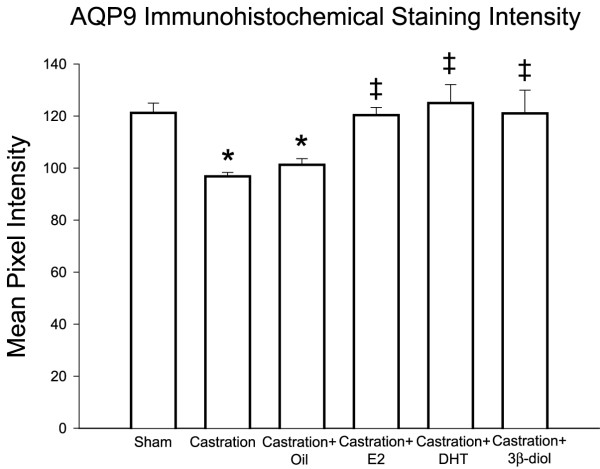
**AQP 9 immunohistochemical staining intensity quantification in the rat efferent ductules**. Castration significantly reduced the AQP9 expression in the efferent ductules compared with controls. Similar result was found in the corn-oil treated group. 3β-diol, as well as estradiol and DHT, restored the efferent ductules AQP9 staining intensity to control level. Values represent mean ± SEM; n = 3 in each group; (*) = P ≤ 0,05 when compared to control group; (‡) = P ≤ 0,05 when compared to castrated group.

In control rats, AQP9 was expressed in efferent ductule epithelium, but restricted to the microvillus border of non-ciliated cells (Fig. 3). Epithelial ciliated cells, as well as the peritubular cells and components of the intertubular tissue were negative for AQP9. Bilateral surgical castration significantly reduced the AQP9 expression by 20% compared with sham-operated controls, as determined by image analysis. Similar result was found in castrated animals that received corn oil. Treatment with estradiol and DHT, as well as 3β-diol, restored staining intensity to control level in the ductule epithelium (Fig. 4).

**Figure 5:**
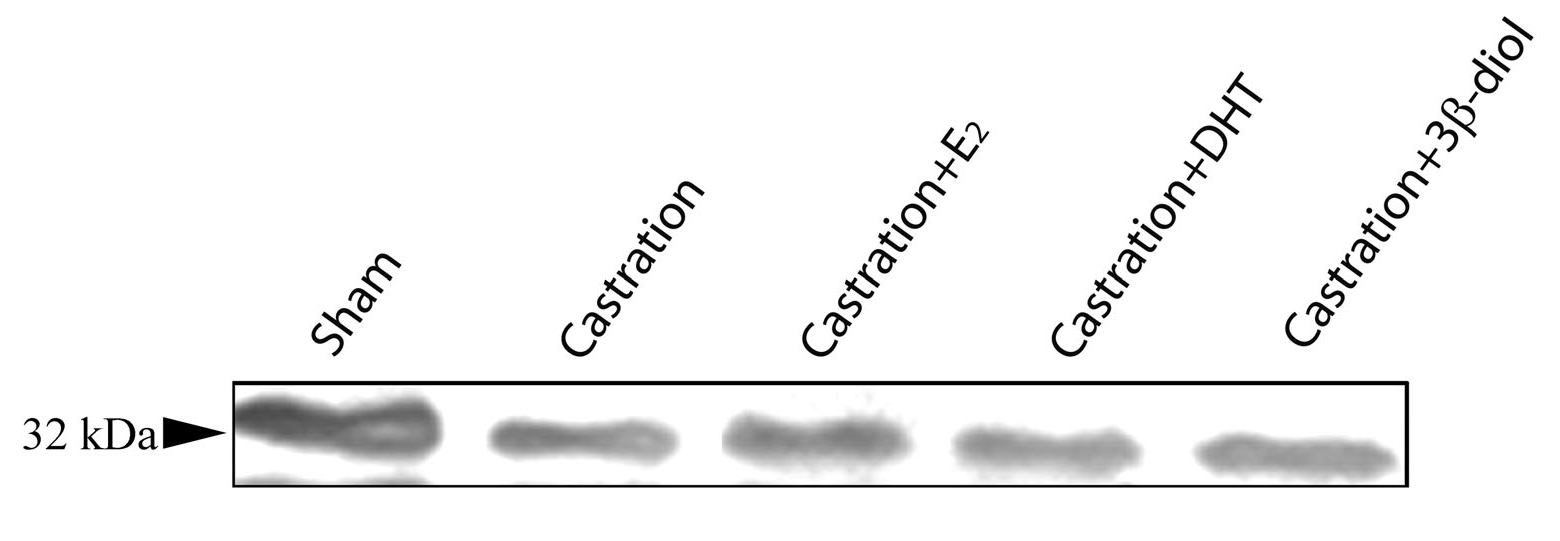
**Western blotting analyses of AQP9 protein extracted from rat efferent ductules**. Compared to the sham control, castration induced a reduction in the 32 kDa AQP9 protein. Treatment of castrated rats with estradiol (E2), DHT or 3β-diol showed partial upregulation of AQP9.

The results were confirmed by Western blotting assay. AQP9 was detected in extracts prepared from rat efferent ductules as a protein band of 32 kDa (Fig. 5), similar to data previously shown for rat genital tract [19]. Comparatively the AQP9 expression was decreased after castration (60%) and greatly recovered after estradiol (70% increase), DHT (52% increase) and 3β-diol (66% increase) replacement (Fig. 5).

## Discussion

In the present study we investigated the relative contributions of estradiol, DHT, and its metabolite 3β-diol, in the regulation of AQP9 expression in adult rat efferent ductules. The data show that 3β-diol was equal in its ability to maintain AQP9 to that observed with estradiol and DHT. These results confirm our previous investigation of AQP9 regulation [16], but add to the growing body of data supporting a steroid hormonal role for 3β-diol in the male reproductive system. Although little is known regarding the physiology of this androgen metabolite, in the present study it was capable of upregulating AQP9, indicating that 3β-diol may serve as an additional ligand for estrogen receptors found in the efferent ductules [40]. The tissue selectivity of 3β-diol action is supported by the fact that the seminal vesicle/coagulating gland weight and blood testosterone or estradiol levels were not affected by 3β-diol treatment, in contrast to ventral prostate and epididymis weights, which were significantly increased by this androgen metabolite.

The importance of DHT in maintaining epididymal structure and function is well established [20,21]. However, compared with epididymis, low 5α-reductase activity has been found in the rodent efferent ductules [41,42]; and AR appears to be expressed at lower levels than in the epididymis [43]. Thus, it is unlikely that DHT resupplementation after castration was able to directly restore the AQP9 staining. If DHT was acting directly through its high affinity binding to AR, then testosterone alone should also have modulated AQP9 expression. Instead, the overall data suggests that it is the DHT metabolite, 3β-diol, that is active in efferent ductules. This conclusion is supported by the following observations:

a) It has been shown that 3β-diol may have hormonal activity, not acting through the AR, but rather as a ligand for both ERα and ERβ.

b) 3β-diol has higher affinity for ERβ [31], which is abundant in the efferent ductule epithelium [40].

c) In human testis, the 3β-diol concentration is higher than DHT and estradiol [44,45]. It is reasonable to postulate that high concentrations of this metabolite may enter the lumen of efferent ductules.

d) The existence of this estrogenic DHT metabolite has raised the possibility that estradiol may not be the major estrogen in males [29]. For instance, in the prostate there is a growing body of evidence that 3β-diol, acting through ERβ, may regulate important physiological events [26,28,32,46].

e) Although the targeted disruption of ERβ has not resulted in alterations of the male reproductive phenotype [47], the aromatase knockout mouse (ArKO), which lacks estradiol and estrone, provides the strongest data in support of 3β-diol having hormonal activity in the male tract. In ArKO mice, spermatogenesis appears normal at first, without efferent ductule abnormalities or disruption in fluid reabsorption, which are observed in the ERα knockout [40,48]. Because estrogen receptors are expressed in the efferent ductule epithelium [40], and are likely expressed in the ArKO mouse, it is reasonable to hypothesize that other ligands, such as 3β-diol, are capable of binding ER for maintenance of normal physiological functions, in the absence of estradiol.

f) Also noteworthy is the fact that 3β-diol stimulates ERβ induced transcriptional activity equal to the cognate ligand estradiol, and the transcriptional selectivity of 3β-diol for ERβ is much greater than its binding selectivity [30,46].

g) AQP9 expression in developing rats progressively increases and reaches adult levels by 4 weeks post-natal [9]. At this time, testosterone is not the predominant androgen, but there are high levels of DHT and its metabolites [24,49]. Thus, 3β-diol could be the alternative modulator of AQP9 expression even during the pre-pubertal period.

These data are in agreement with those of Badran and Hermo (2002), who concluded that a luminal factor coming from the testis, but not testosterone *per si*, was involved in the modulation of AQP9 in the initial segment epididymis. As shown more recently, targeted disruption of ERα resulted in a small reduction of AQP9 in the male tract [17], providing further support for the participation of another testosterone metabolite in the regulation of AQP9. It is also relevant that female tissues show a similar regulation of AQP9 by androgen metabolites, such as estradiol [50]. Taken together, these data indicate that the underlying mechanisms for AQP9 regulation are complex and likely involve multiple androgen metabolites.

## Conclusion

In conclusion, this is the first evidence that 3β-diol has biological activity in the efferent ductules. Data presented here support the hypothesis that DHT regulation of AQP9 expression in efferent ductules is likely to be mediated by its reduction to 3β-diol and that this androgen metabolite may play a role in luminal fluid homeostasis.

## Competing interests

The author(s) declare that they have no competing interests.

## Authors' contributions

PPL and AGO carried out the experiments and wrote the first draft of the manuscript. AMR assisted with the radioimmunoassay. EK assisted with the Western Blotting assay. GABM provided valuable laboratory assistance and helped to design the study. RAH helped with study design and critically revised the manuscript. CAO served as PI on this study, carried out the study design and wrote the final draft. All authors read and approved the final manuscript.
